# Extra-Corporeal Membrane Oxygenation in Pregnancy

**DOI:** 10.3390/jcm13061634

**Published:** 2024-03-13

**Authors:** Tatsiana Romenskaya, Yaroslava Longhitano, Aman Mahajan, Gabriele Savioli, Antonio Voza, Manfredi Tesauro, Christian Zanza

**Affiliations:** 1 Department of Physiology and Pharmacology, Sapienza University of Rome, 00185 Rome, Italy; tatsiana_romenskaya@yahoo.it; 2 Department of Anesthesiology and Perioperative Medicine, University of Pittsburgh Medical Center (UPMC), Pittsburgh, PA 15261, USA; lon.yaro@gmail.com (Y.L.);; 3 Department of Emergency Medicine, IRCCS Foundation Polyclinic San Matteo, 27100 Pavia, Italy; 4 Department of Emergency Medicine, IRCCS-Humanitas Research Hospital, Rozzano, 20089 Milan, Italy; antonio.voza@humanitas.it; 5 Department of Systems Medicine, University of Rome “Tor Vergata”, 00133 Rome, Italy; 6 Geriatric Medicine Residency Program, University of Rome “Tor Vergata”, 00133 Rome, Italy; 7 Italian Society of Prehospital Emergency Medicine (SIS 118), 74121 Taranto, Italy

**Keywords:** ECMO, pregnancy, H1N1, COVID-19, ARDS, cardiac arrest

## Abstract

Extracorporeal membrane oxygenation (ECMO) is a cardiac or pulmonary function support system that is used in cases of refractory organ failure in addition to conventional treatment. Currently, Level I evidence is not yet available, which reflects improved outcomes with ECMO in pregnant women, the use in pregnancy should be indicated in selected cases and only in specialized centers. We searched articles in the most important scientific databases from 2009 until 31 December 2023 consulting also the site ClinicalTrials.com to find out about studies that have been recently conducted or are currently ongoing. We matched the combination of the following keywords: “ECMO and pregnancy”, “H1N1 and pregnancy”, “COVID-19 and pregnancy”, “ARDS and pregnancy”, “ECMO and pregnancy AND (cardiac arrest)”. We selected the following number of articles for each keyword combination: “ECMO and pregnancy” (665 articles); “ECMO and influenza H1N1” (384 articles); “pregnancy and influenza H1N1” (1006 articles); “pregnancy and ARDS” (2930 articles); “ECMO and pregnancy and ARDS and influenza H1N1” (24 articles); and “[ECMO and pregnancy AND (cardiac arrest)]” (74 articles). After careful inspection, only 43 papers fitted our scope. There are two types of ECMO: venous-venous (VV-ECMO) and venous-arterial (VA-ECMO). The first-one is necessary to cope with severe hypoxia: oxygen-depleted blood is taken from the venous circulation, oxygenated, and carbon dioxide removed from the extracorporeal circuit and returned to the same venous system. The VA-ECMO is a type of mechanical assistance to the circulatory system that allows to put the failing organ at rest by ensuring adequate oxygenation and systemic de-oxygenation, avoiding multi-organ failure. The main indications for ECMO support in pregnant women are cardiogenic shock, acute respiratory distress syndrome (ARDS), pulmonary embolism, and eclampsia. There are also fetal indications for ECMO, and they are fetal distress, hypoxic-ischemic encephalopathy (HIE), and twin-to-twin transfusion syndrome (TTTS). Until now, based on the outcomes of the numerous clinical studies conducted, ECMO has been shown to be a successful therapeutic strategy in cases where medical treatment has been unsuccessful. In well-selected pregnant patients, it appears to be safe and associated with a low risk of maternal and fetal complications. The aim of this review is to report the main properties of ECMO (VV and VA) and the indications for its use in pregnant women.

## 1. Introduction

Extracorporeal membrane oxygenation (ECMO) is a cardiac or pulmonary function support system that is used in cases of organ failure refractory to conventional treatment. Over the past decades, the use of this therapeutic strategy has increased considerably, due in part to the simplification of the technology used, also in the pregnant women population [1,2]. Pregnant women are a special population who require specific attention, particularly in intensive care medicine. It is well known that a pregnant woman undergoes physiological changes that can have an impact on the cardiovascular and respiratory systems, greatly increasing the risk of complications in the event of a serious illness.

The ECMO treatment strategy is also being increasingly used among women of fertile age or pregnant women due to the fact that cardiovascular risk factors are constantly growing. In addition, the H1N1 influenza and SARS-CoV-2 pandemics have revealed that ECMO can be used in pregnant patients with refractory hypoxemia [3,4,5]. Sharma et al. (2015) published a review on the use of ECMO in pregnant women and in the postpartum period. The authors examined clinical cases of patients with respiratory distress who subsequently developed cardiorespiratory failure requiring ECMO from 2009 to 2014. In that observational study, overall maternal survival was observed to be 80% and fetal survival was 70% [6].

Palella et in 2023 conducted a systematic review in 306 women, 203 were prepartum at the time of cannulation (66.3%), and 103 were postpartum (33.7%). The result obtained suggested that VV-ECMO in this population could save five out of six mothers (survival > 80%), while fetal mortality was doubled with approximately one-third unfavorable outcomes (fetal survival ca. 67.9%) [7].

There are two types of ECMO: venous-venous (VV-ECMO) and venous-arterial (VA-ECMO). The first modality is necessary to cope with severe hypoxia: oxygen-depleted blood is taken from the venous circulation, oxygenated, and carbon dioxide removed from the extracorporeal circuit and returned to the same venous system [1,8].

On the other hand, veno-arterial ECMO is a type of mechanical assistance to the circulatory system that allows us to put the failing organ at rest by ensuring adequate oxygenation, carbon dioxide removal avoiding multi-organ failure.

The literature highlight that the application of ECMO in pregnancy is safe and practicable when performed in an experienced center and is generally associated with satisfactory results [9].

The aim of this narrative review is to report the main properties of ECMO (VV and VA) and the indications for its use in pregnant women.

## 2. Materials and Methods

We conducted a search on major available scientific databases such as Cochrane Controlled Trials Register, Cochrane Library, Medline/Pubmed, Google Scholar, Ovid/Wiley and ClinicalTrials.com for recently conducted or currently ongoing trials. We matched the following keywords: “ECMO and pregnancy”, “Influenza H1N1 and pregnancy”, “COVID19 and pregnancy”, “ARDS and pregnancy”, “ECMO and influenza H1N1”, “ECMO and pregnancy and ARDS and influenza H1N1”, and “ECMO and pregnancy AND cardiac arrest”.

## 3. Results

For every matched group, we found 665 articles for “ECMO and pregnancy” 384 for “ECMO and influenza H1N1”; 1006 for “pregnancy and influenza H1N1”; 2930 for “pregnancy and ARDS”; 24 for “ECMO and pregnancy and ARDS and influenza H1N1” and 74 for “ECMO and pregnancy and cardiac arrest”. After careful inspection, we included in our review 43 articles.

## 4. Discussions

Extracorporeal membrane oxygenation uses a specialized external circuit that temporarily supports the lungs and/or heart, thus permitting to support these vital organs and avoiding multi-organ failure. Principally, ECMO can be divided into two main modes: veno-venous (VV) and veno-arterial (VA).

What VV and VA ECMO both have in common is that they are intended to improve blood oxygenation and remove waste products, such as carbon dioxide, from the patient’s blood. In this way, oxygen-rich blood will be transported to all vital organs, ensuring proper functioning [10].

The main difference between these two systems is that with VV ECMO, de-oxygenated blood is withdrawn from the venous system but is also returned via the venous system, making it possible to put the lung at rest and accelerate healing in cases of severe respiratory failure and/or inflammation.

VA ECMO, on the other hand, not only provides oxygenation support but also gives circulatory support by bypassing the heart and returning oxygenated blood directly to the arterial system. This can be fundamental in cases of severe heart failure or cardiogenic shock [11,12].

Therefore, VV ECMO provides respiratory support, whereas VA ECMO provides both respiratory and cardiovascular support [13,14].

During the gestational period, a woman’s body undergoes numerous significant physiological changes. The blood volume increases between 30 and 50% to almost 4000 mL, which leads to a significant increase in cardiac output and oxygen consumption. Cardiac output increases by 30–50% (from 4.5 to about 6 L/min) and is due to both the increase in stroke volume and the increase in heart rate. These changes may have an impact on the selection of ECMO cannulation sites, flow rates, and anticoagulation management, as altered physiology may affect blood flow and coagulation factors, as pregnancy will lead to a prothrombotic state but with dilution thrombocytopenia [6].

In addition, the presence of an unborn child adds an extra level of complexity to the decision-making process. In the case of severe maternal hypoxia, the fetus can tolerate it for a long time thanks to physiological adaptations such as the leftward shift of the fetal oxygen-hemoglobin dissociation curve, fetal polycythemia, and the redistribution of fetal blood flow to vital organs. Maternal ECMO support must balance the needs of the mother and the fetus.

Factors such as gestational age, fetal well-being, and the potential effects of ECMO on fetal development and viability must be attentively considered [15].

### 4.1. Indications for ECMO Support

The summary of indications for ECMO (VV and/or VA) in pregnant women and fetuses is represented in Table 1, and they are further described below.

Pregnant women, even in the absence of pre-existing risk factors, can unfortunately present with severe heart failure that can develop into cardiogenic shock. This may be caused by myocardial infarction, myocarditis, peripartum cardiomyopathy, or arrhythmias. In that case, the heart will be unable to provide normal circulation, and consequently, the demands of both mother and fetus cannot be met. ECMO can help provide circulatory and oxygen support while treating the cause at the same time [16]. This type of treatment is indicated, especially in the case of cardiogenic shock refractory to medical therapy; it is not always a destination therapy but only a bridge (it is a bridging device for decision-making).

Acute respiratory distress syndrome (ARDS) is a serious clinical condition with severe respiratory dysfunction. The cause of ARDS in pregnant women may be bacterial or viral pneumonia (H1N1 or SARS-CoV-2), sepsis, or, more rarely, aspiration pneumonia. ECMO takes over when therapeutic strategies such as medical therapy and protective mechanical ventilation have failed [17]. Reviewing the literature, there is no strong evidence that support for VV-ECMO leads to better results in the treatment of severe ARDS. Indications for VV-ECMO support during pregnancy in cases of respiratory failure/ARDS are listed in Table 2. VV-ECMO may be considered in ARDS refractory to conventional treatment modalities in centers with a large number of years of experience and a dedicated ECMO team. More data are required to define the role of VV-ECMO in the treatment of ARDS, such as the comparison between protective mechanical ventilation and ECMO in pregnant women. Actually, the scientific evidence in this regard is even more limited in the population of pregnant women. The largest use of VV-ECMO support in pregnancy was observed during pandemics, such as H1N1 influenza in 2009 and the SARS-CoV-2 infection from 2020 to 2021 [18]. Furthermore, in a meta-analysis conducted by Saad et al., it was shown that survival among pregnant women with ARDS secondary to H1N1 was 75% [19].

Pulmonary embolism: Pregnant women are at high thrombotic risk due to physiological changes during the gestational period. As a consequence, pulmonary embolism can lead to severe respiratory failure associated with or without cardiovascular dysfunction. Again, ECMO is the last option where other treatments fail and will ensure both cardiopulmonary support and adequate anticoagulant therapy [20].

Cardiac arrest: In cases of cardiac arrest in pregnant women, there is no evidence to support routine VA ECMO use to this day. Despite that, this strategy may be considered in pregnant women in cardiac arrest from reversible causes (e.g., bupivacaine intoxication) after conventional cardiopulmonary resuscitation according to ALS for at least 10 min without success [17].

Eclampsia: Eclampsia is represented by unexplained, generalized convulsive seizures in patientsdiagnosed with pre-eclampsia. In a case series of 1276 patients with pre-eclampsia or severe eclampsia, 3% developed pulmonary edema [21,22]. This very rarely evolves into cardiovascular collapse and acute respiratory distress. ECMO may support a refractory eclamptic crisis or severe cardiovascular compromise [23].

Fetal Indications for ECMO: Fetal distress [24] is due to the severe impairment in oxygenation and perfusion during the intrauterine period, may be caused by the severe clinical condition of the mother. Hypoxic-ischemic encephalopathy (HIE): HIE is a severe neurological disorder in neonates due to severe oxygen deficiency that can develop into severe hypoxia during labor. In 2011, Allen et al. published a paper in which, among the possible treatments for this severe condition, the authors proposed the use of ECMO to ensure the provision of oxygenation and circulatory support to the fetus, allowing time for potential recovery and minimizing further neurological damage [25]. No further scientific evidence is available for this therapeutic strategy.

Twin-to-twin transfusion syndrome (TTTS): This problem happens in about 15% of identical twin pregnancies. This single placenta contains blood vessels going from one baby to the other. This is usually balanced but is unbalanced in the setting of TTTS. In TTTS, blood from the smaller “donor” twin is transferred to the larger “recipient” twin through interconnecting vessels, causing an unequal exchange of blood. The recipient twin is larger and is at risk for heart failure because he received too much blood from the donor twin. ECMO may be used to support the cardiovascular system of the affected twin, while treatment options, such as fetoscopy laser therapy or selective feticide are considered [26].

### 4.2. What Does the ECMO Circuit Consist of?

#### 4.2.1. Veno-Arterial ECMO

**Figure 1:**
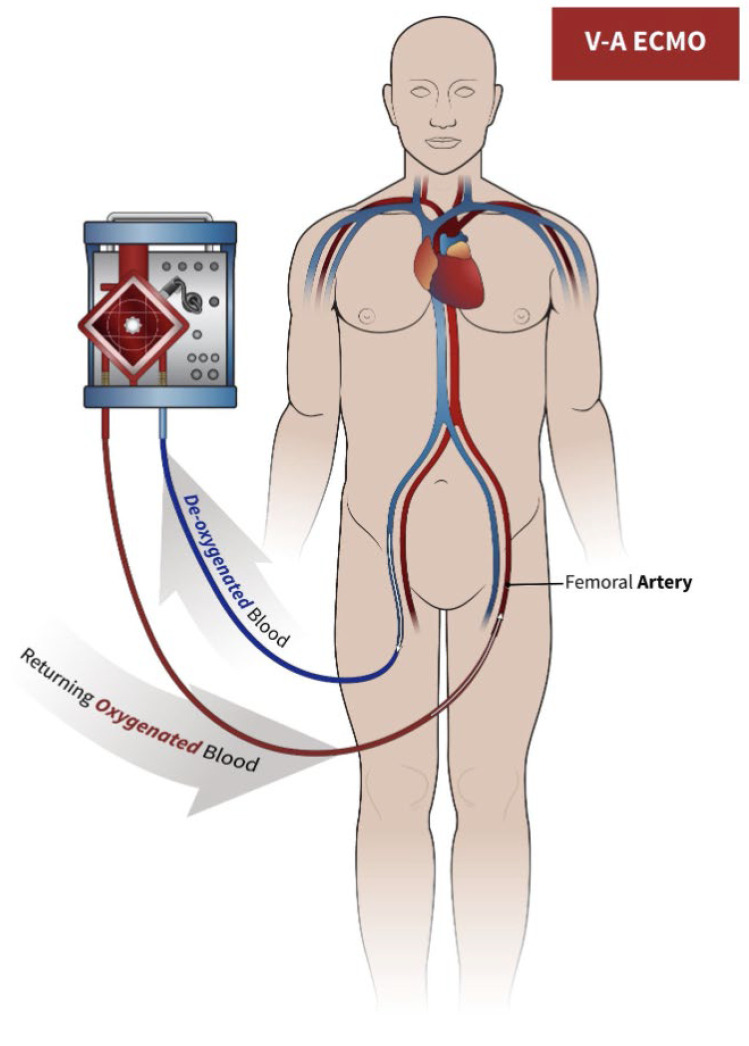
The circuit for veno-arterial extracorporeal membrane oxygenation (VA-ECMO). Blood is drawn into the ECMO circuit (blue arrow) from venous lumen, and after oxygenation and exposure to sweep gas, it is returned to the mother (red arrow) through the arterial lumen.

The V-A ECMO circuit consists of two cannulas, a pump, pipes, an oxygenator, and a warmer (see Figure 1). The system takes blood from the venous district with the in-flow cannula, and the pump and tubing take it into an oxygenator.

It is easy to place, and no monitoring is needed for invasive placement (no need for an echocardiogram or even a fluoroscope). It can be placed percutaneously or surgically and is a system that allows us to support both ventricles, but most pregnant patients requiring VA ECMO do not require cardiac surgery. Central cannulation occurs mainly during cardiac surgery and is required by those patients who do not tolerate weaning from cardiopulmonary bypass. In that case, the cannulas are placed in the right atrium and proximal aorta.

The oxygenator is a ‘box’ made of many channels (made of polymethylpentene or polypropylene) that allow the blood to exchange O_2_–CO_2_ with the air flow passing against the current. It is possible to adjust the intensity of the O_2_–CO_2_ exchange by moving the gas sweep. After passing through the oxygenator, the blood passes through the heat exchanger to make it warmer or colder, and then enters the patient’s arterial circulation (usually in the thoracic artery) [27].

The effect of VA-ECMO on uterine circulation is unknown, and if this form of support is needed in a life-sustaining pregnancy, continuous fetal monitoring is recommended [5]. Patients should always be in lateral position (especially after 20 weeks of pregnancy) because uterine compression on the inferior vena cava and aorta can disturb blood flow in cases of VA-ECMO (not so much in VV-ECMO, since most patients will have a single double-lumen cannula in the right jugular vein) [5].

#### 4.2.2. Veno-Venous ECMO

On VV-ECMO Oxygen-depleted blood is taken from the venous circulation, oxygenated, and carbon dioxide removed from the extracorporeal circuit and returned to the same venous system. A venous drainage cannula is usually placed in the right internal jugular vein, and a venous cannula is used for the re-infusion of oxygenated blood into the common femoral vein. Through the cannula placed in the internal jugular vein, the blood is drained and conveyed to the membrane oxygenator, where it is oxygenated by removing carbon dioxide. Subsequently, the blood is reinfused into the venous circulation near the right atrium through the cannula positioned in the common femoral vein. When the flow through a single drainage cannula is insufficient, especially in severe respiratory failure, a second drainage cannula is placed in the other common femoral vein.

Within the extracorporeal circuit, the blood passes through an oxygenator and a heat exchanger that heats it before returning to the patient. The fresh air (sweep gas) and oxygen are mixed in a mixer before this gas is exposed to the blood through a semi-permeable membrane [5].

**Figure 2:**
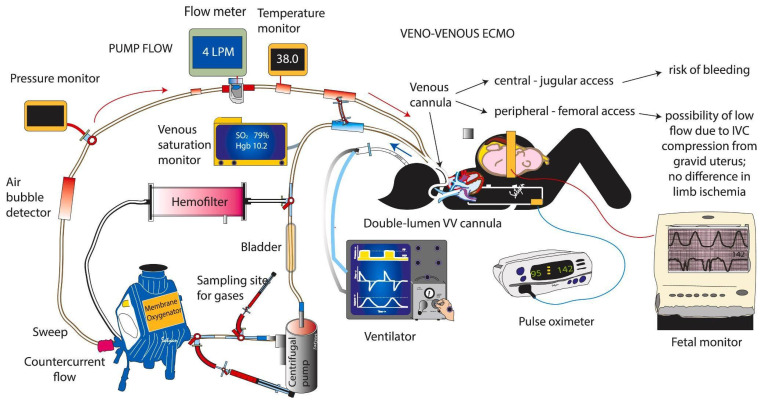
The circuit for veno-venous extracorporeal membrane oxygenation (VV-ECMO) and monitoring and support in a pregnant woman. VV-ECMO can be conducted using two cannuales (jugular and/or femoral) or with one double-lumen cannula, as shown in the figure. Blood is drawn into the ECMO circuit (blue arrow) from one lumen, and after oxygenation and exposure to sweep gas, it is returned back to the infant (red arrow) through the second lumen. The mother is monitored with frequent blood gases and pulse oximetry. The fetal heart rate is closely monitored. [29] Figure courtesy of Satyan Lakshminrusimha.

Oxygenation is determined by the capacity of the circuit. The oxygen content of the blood depends on the level of hemoglobin, the partial pressure of oxygen, the dissociation curve of oxyhaemoglobin, and, to a very small part, the amount of dissolved oxygen. Once oxygenation and carbon dioxide clearance have been improved by ECMO, ultra-protective mechanical ventilation can be used to further reduce iatrogenic lung damage from high pressures and high oxygen concentrations [27]. In a recent clinical trial conducted by Comb et al. [28], two groups of patients with severe ARDS were analyzed: the first group received immediate VV ECMO treatment (ECMO group), and the second group underwent conventional treatment (control group). The result showed that the best benefit of VV ECMO was the reduction of ventilator-induced iatrogenic lung injury (VILI) (Figure 2).

### 4.3. Weaning from ECMO

The start of weaning from extracorporeal circulation can be considered when the underlying pathological process for which treatment was initiated is sufficiently resolved.

The weaning from VA ECMO occurs when the patient’s hemodynamics begin to improve by checking the intrinsic cardiac performance using transthoracic ultrasound. In that case, we start decreasing flow support, lowering it to 1–1.5 L/min, and monitoring the patient’s cardiac response [30].

The weaning from VV ECMO will start according to the criteria based on recovery of respiratory function in terms of gas exchange, respiratory mechanics measurements, and chest imaging. A commonly accepted and considered safe approach involves the incremental reduction of gas and blood flows until gas flow is completely stopped. The patient is then observed for a variable period of time in the absence of extracorporeal gas exchange (e.g., 30 min or more) to ensure the stability of the patient’s condition and then proceed to decannulation [5].

### 4.4. Complications & Contraindications

As has been described in previous sections, ECMO can indeed, in some cases, be a life-saving therapy, but this therapeutic strategy is not entirely without complications. These complications occur mainly in pregnant women due to the physiological changes that occur during pregnancy. The main complications include bleeding, infection, thrombocytopenia, neurological events, Harlequin syndrome, and thrombosis of the ECMO circuit (see Table 3).

Throughout the use of ECMO, systemic anticoagulation must be administered to prevent thrombosis of the circuit, but this exposes the patient to an increased risk of bleeding [30] and may be a leading contributor to maternal mortality and morbidity [31,32].

Two new anticoagulation strategies in order to reduce the risk of bleeding during ECMO treatment, such as Nafamostat Mesilate (NM) and Bivalirudin, are currently mentioned in the literature. The research group of Lee JH et al. published a retrospective review in 2021 in which they analyzed the aPTT values of 16 patients undergoing ECMO. Seven of these patients were switched to NM after first using heparin as an anticoagulation agent, and nine received only NM. The result obtained is statistically significant (*p* = 0.010) and suggests that the aPTT value of the patients in the NM group was lower than that at the ECMO site (73.57 vs. 79.25 s) [33].

Sanfilippo et al. recently published a paper supplementing with a recent update meta-analysis on strategies to compare anticoagulation, heparin or bivalirudin, in patients undergoing ECMO. What emerges is that bivalirudin may provide survival benefits and reduce thrombosis in a subgroup of adult patients undergoing ECMO [34].

A randomized controlled trial is urgently needed for patients supported by ECMO, especially pregnant women.

As is well known, during pregnancy, a prothrombotic state occurs as a result of physiological changes. Even during ECMO support, a prothrombotic state is present due to the interaction of the extracorporeal circuit with the circulatory system and the activation of the extrinsic coagulation pathway. Throughout the use of the extracorporeal system, anticoagulant drugs must be administered to prevent clotting complications and maintain the delicate equilibrium between preventing thrombotic events and avoiding excessive bleeding [35]. Thus, a pregnant woman undergoing ECMO may develop ‘ECMO-associated coagulopathy’. The latter is mainly due to three mechanisms: hemodilution, platelet dysfunction (both in terms of thrombocytopenia and malfunctioning), and acquired Von Willebrand disease, which can develop within one day of starting ECMO. Ultra-specialized ECMO centers target hemoglobin above 7–8 g/dL and platelets > 50,000/mm^3^.

Much more rarely than coagulation alterations, neurological complications such as ischaemic stroke or hemorrhagic stroke have been observed. The rate of brain injury can be as high as 19% in VA ECMO and 11% in VV ECMO [36]. Although ischaemic complications and anoxic injury are typically more common in VA ECMO, the incidence of intracranial hemorrhage (5% to 10%) appears to be similar in both forms of cannulation.

Harlequin syndrome is a complication that can occur during a VA ECMO due to the competitive flow between the blood flow of the native heart (orthograde flow) and the flow of the ECMO (retrograde flow). The strength of the orthograde flow is such that it restricts the retrograde flow from reaching the head, so that two different perfusion circuits are generated: in the lower part of the body, there is perfusion by the ECMO with oxygenated blood, so that the organs and skin complexion appear normal. In the upper part of the body, blood flow from the heart restricts the arrival of retrograde flow, with the presence of cyanosis in the upper half of the body and signs of tissue hypoxia, especially in the brain and heart. It appears more frequent when the patient also has severe hypoxaemia (pulmonary oedema, ARDS, etc.), with recovery of cardiac function being more rapid than recovery of pulmonary function, as the blood passing from the lungs is not oxygenated by either ECMO or residual pulmonary function [37]. Good saturation at the right radial artery greatly reduces the risk of a Harlequin syndrome; on the other hand, in the case of hypoxia in the right radial artery, it appears essential to check the ECMO saturation to see whether two different circulators are being generated (generally there are differences in saturation of more than 10–15%).

Unfortunately, most cases of VV-ECMO use have been described in the available literature. A recent meta-analysis by Moore et al. summarized the available evidence on ECMO support during pregnancy [3]. The authors had identified 45 cases, of which only four referred to the use of VA ECMO. Maternal and fetal survival after VA-ECMO were 77.8% and 65%, respectively. Instead, according to another systematic review, the pooled prevalence of maternal and fetal survival was 77.2% and 69.1%, respectively, and the most common maternal complication was bleeding (37.2%) [38].

Ong et al. published a systematic review in 2020 of 90 clinical cases where pregnant or postpartum patients necessitated ECMO support. The result of the statistical analysis was that in the 97 pregnant or postpartum patients, the maternal and neonatal survival rates were 90.7% (88/97) and 83.3% (80/96, one undocumented), respectively. Among the complications arising during ECMO support, hemorrhage outweighed the others, such as infections, atrial fibrillation, renal impairment, and generalized myopathy. Survival was better for cardiac than respiratory indications [39].

Furthermore, ECMO can have negative effects on a fetus, such as a delay in normal physiological development and neurodevelopment, and therefore close fetal monitoring during extracorporeal support is necessary [40].

In 2016, Saad et al. published the results of the meta-analysis that demonstrated neonatal outcomes in terms of the rate of live birth were 70% (95% CI 43.7–95.2) in women undergoing VV-ECMO. In addition, infants born alive to mothers on VV-ECMO had low Apgar scores (0–6) and respiratory failure, necessitating respiratory support in the ICU [41]. Regarding the contraindications for ECMO support, there are absolute contraindications, including ongoing hemorrhagic shock, aortic dissection, and severe aortic valvular insufficiency [27].

## 5. Conclusions

Until now, based on the outcomes of the numerous clinical studies conducted, ECMO has been shown to be a successful therapeutic strategy in cases where medical treatment has been unsuccessful [42,43].

ECMO provides short-term relief to vital organs such as the heart and lungs, allowing time for them to heal. In well-selected pregnant patients, it appears to be safe and associated with a low risk of maternal and fetal complications.

Notwithstanding the excellent results of this therapeutic strategy, the risks and both maternal and fetal complications associated with ECMO also must be taken seriously.

Before starting extracorporeal support, a meticulous multidisciplinary and ethical evaluation is required to determine the best course of treatment for each clinical case. But thanks to the clinical studies currently underway and those that will be carried out in the future, it will be possible to improve this technique in such a specific population as pregnant women in order to avoid both short- and long-term complications for maternal and fetal health.

Our review has a few limitations, primarily that it is a narrative review. Surely, further research in this sensitive field would be useful. It would also be useful to write a systematic review encompassing all the topics discussed in this article by the authors.

## Figures and Tables

**Table 1:** Indications for ECMO support (Veno-Arterial and Veno-Venous) during pregnancy.

**Maternal Indication**	Cardiogenic Shock	Acute Respiratory Distress	Eclampsia	Pulmonary Embolism
**Fetal Indication**	Twin to Twin Transfusion Syndrome	Hypoxic- Hischemic encephalopaty	Fetal Distress	

**Table 2:** Indications for Veno-Venous-Extra Corporeal Membrane Oxygenation support during pregnancy during respiratory failure/acute respiratory distress syndrome.

Indications for Veno-Venous-Extra Corporeal Membrane Oxygenation Support during Pregnancy in Case of Respiratory Failure/Acute Respiratory Distress Syndrome
- Severe hypoxia and respiratory failure. - Pressure arterial Oxygen/Fraction inspiratory Oxygen ratio less than 100 with inspired fraction of oxygen ≥ 0.9 and Positive End Espiratory Pressure ≥ 10 cm H_2_O despite optimal ventilator support and use of usual adjunctive methods (e.g., cisatracurium, recruitment maneuvers, prone ventilation, and adequate PEEP). - Hypercapnia with severe respiratory acidosis with - Respiratory Rate > 35 breaths/min.

**Table 3:** ECMO VV/VA complications.

Bleeding	Due to “ECMO-Associated Coagulopathy” the Etiology Is Multifactorial, Including Anticoagulation, Thrombocytopenia and Acquired Von Willebrand Disease
Infection	Should be administered a prophylactic antibiotic (low evidence).
Thrombo- cytopenia	The goal is to maintain hemoglobin above 7 to 8 g/dL and platelets above >50,000/mm^3^.
Neurological complications	Ischemic and hemorrhagic strokes and anoxic brain injury. It is more common in Veno-Arterial Extra Corporeal Membrane Oxygenation than in Veno-Venous Extra Corporeal Membrane Oxygenation (19% vs. 11%, respectively).
Harlequin syndrome	Regional changes in skin coloration, with cyanosis of the upper portions of the body and a pinkish complexion of the lower regions.
Thrombosis of ECMO circuit	Can be prevented with systemic anticoagulation.

## Data Availability

Not applicable.

## Abbreviations

ALS = advanced life support; ARDS = acute respiratory distress syndrome; ECMO = extracorporeal membrane oxygenation; VA = veno-arterial; VV = veno-venous; HIE = hypoxic-ischemic encephalopathy; TTTS = twin-to-twin transfusion syndrome; RCP = resuscitation cardiopulmonary; VILI = ventilator-induced lung injury; PEEP = positive end-expiratory pressure.
